# Supplementary material for: The Study of pH Effects on Phase Transition of Multi-Stimuli Responsive P(NiPAAm-co-AAc) Hydrogel Using 2D-COS
Source: Polymers (Basel). 2021 Apr 29;13(9):1447. doi: 10.3390/polym13091447 (PMC8125589; doi:10.3390/polym13091447)
Supplement: Supplementary file 1 [file polymers-13-01447-s001.zip › polymers-1175759-supplementary.pdf]

Supporting Information

The study of pH effects on phase transition of multi-stimuli responsive P(NiPAAm-*co*-AAc) hydrogel using 2D-COS

Yeonju Park [1], Minkyoung Kim [2], Hae Jin Chung [2], Ah Hyun Woo [2], Isao Noda [3] and Young Mee Jung [1,2,*]

[1] Kangwon Radiation Convergence Research Support Center, Kangwon National University, Chuncheon 24341, Korea; yeonju4453@kangwon.ac.kr (Y.P.), ymjung@kangwon.ac.kr (Y.M.J.)

[2] Department of Chemistry, Institute for Molecular Science and Fusion Technology, Kangwon National University, Chuncheon 24341, Korea; alsrud9351@naver.com (M.K.), jhyejin1212@gmail.com (H.J.C.), dndkgus99@naver.com (A.H.W.), ymjung@kangwon.ac.kr (Y.M.J.)

[3] Department of Materials Science and Engineering, University of Delaware, Newark, DE 19716, USA; noda@udel.edu (I.N.)

* Correspondence: ymjung@kangwon.ac.kr; Tel.: +82-33-250-8495

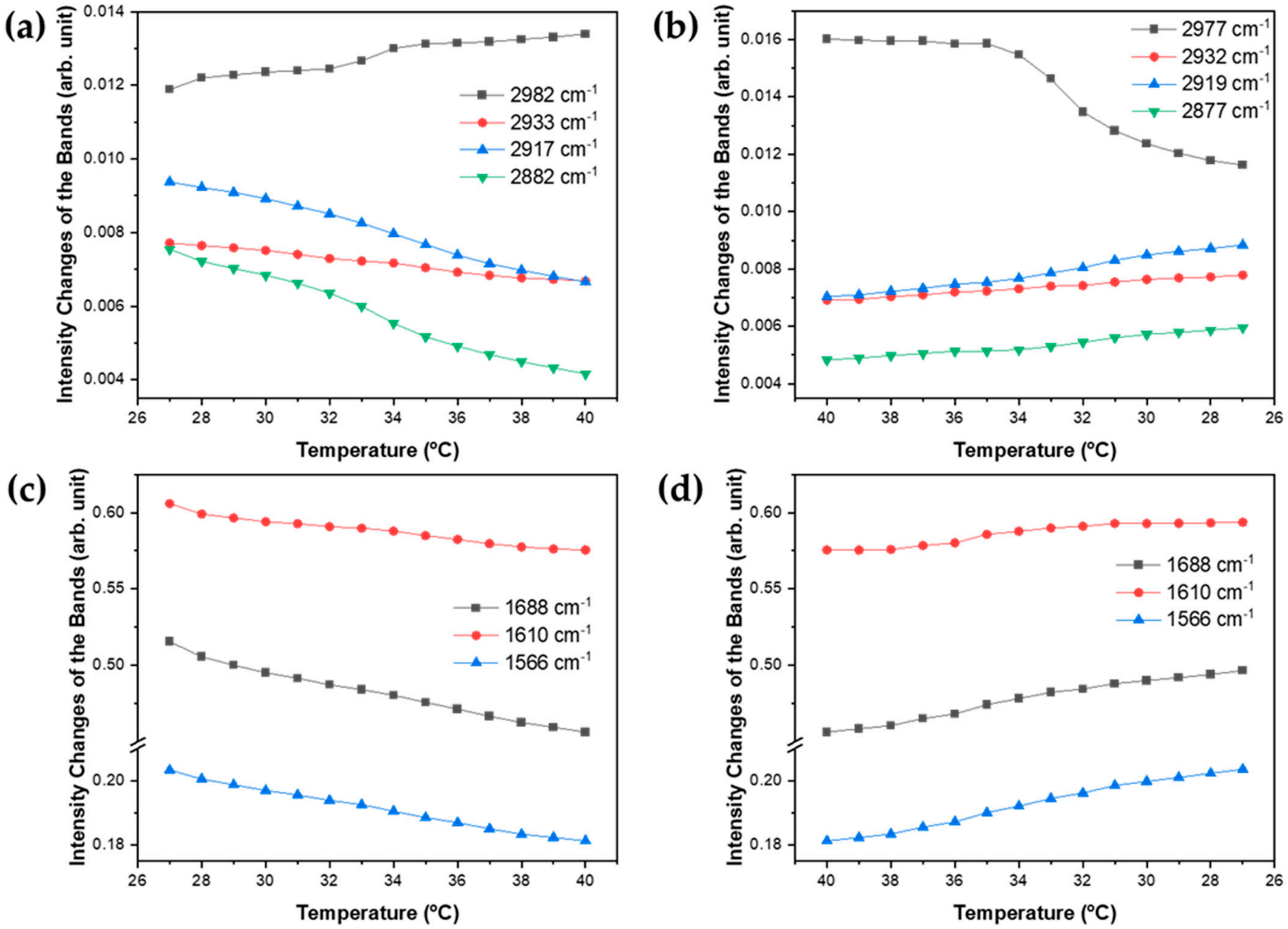

Figure S1. Intensity changes of the bands in two spectral regions (a,b: 3010-2850 and c,d: 1800-1480 cm^{-1}) of P(NPAAm-*co*-AAc) hydrogel at pH4 during the heating (a, c) and cooling (b, d) processes.

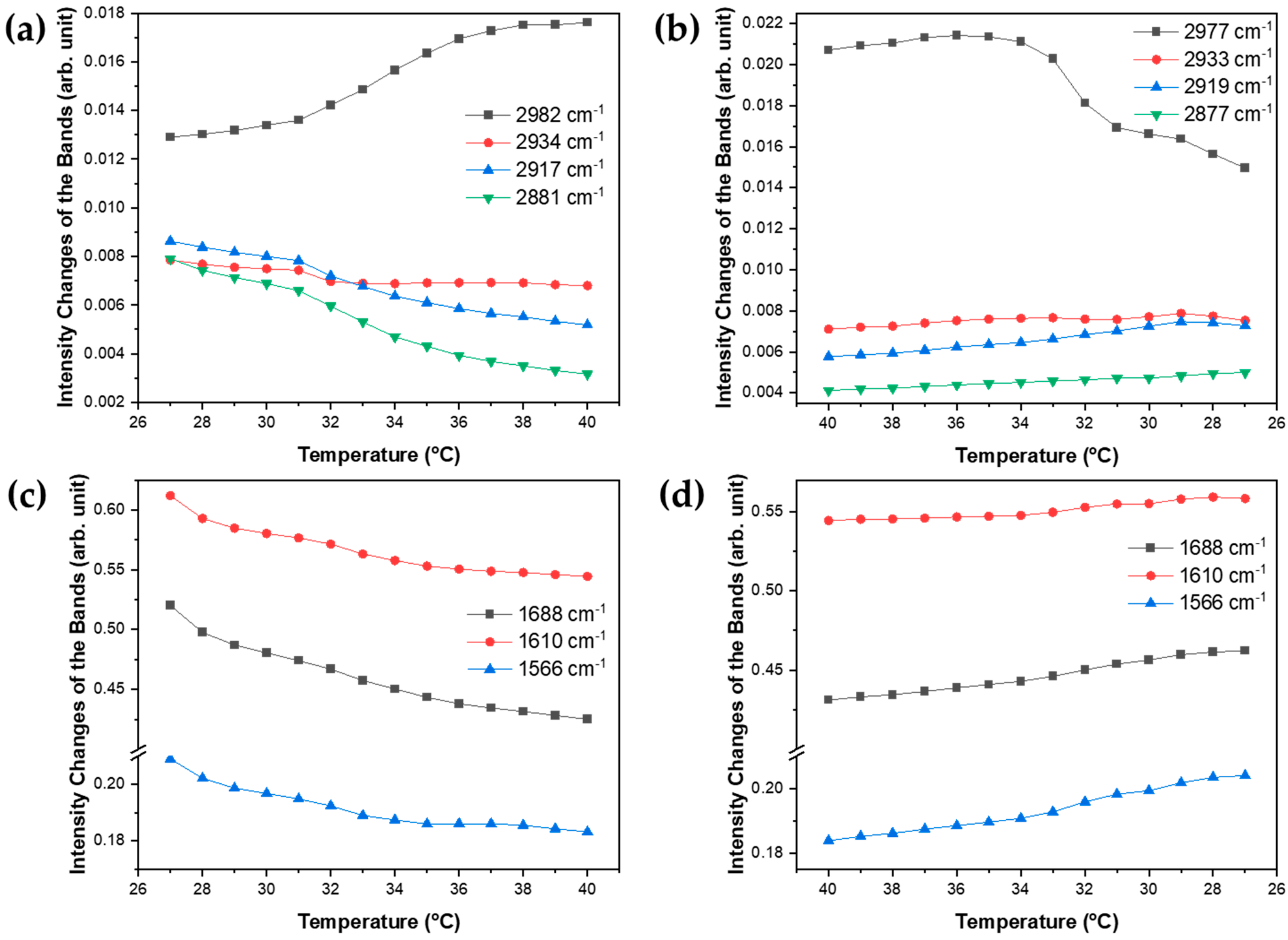

Figure S2. Intensity changes of the bands in two spectral regions (a,b: 3010-2850 and c,d: 1800-1480 cm^{-1})of P(NPAAm-*co*-AAc) hydrogel at pH3 during the heating (a, c) and cooling (b, d) processes.

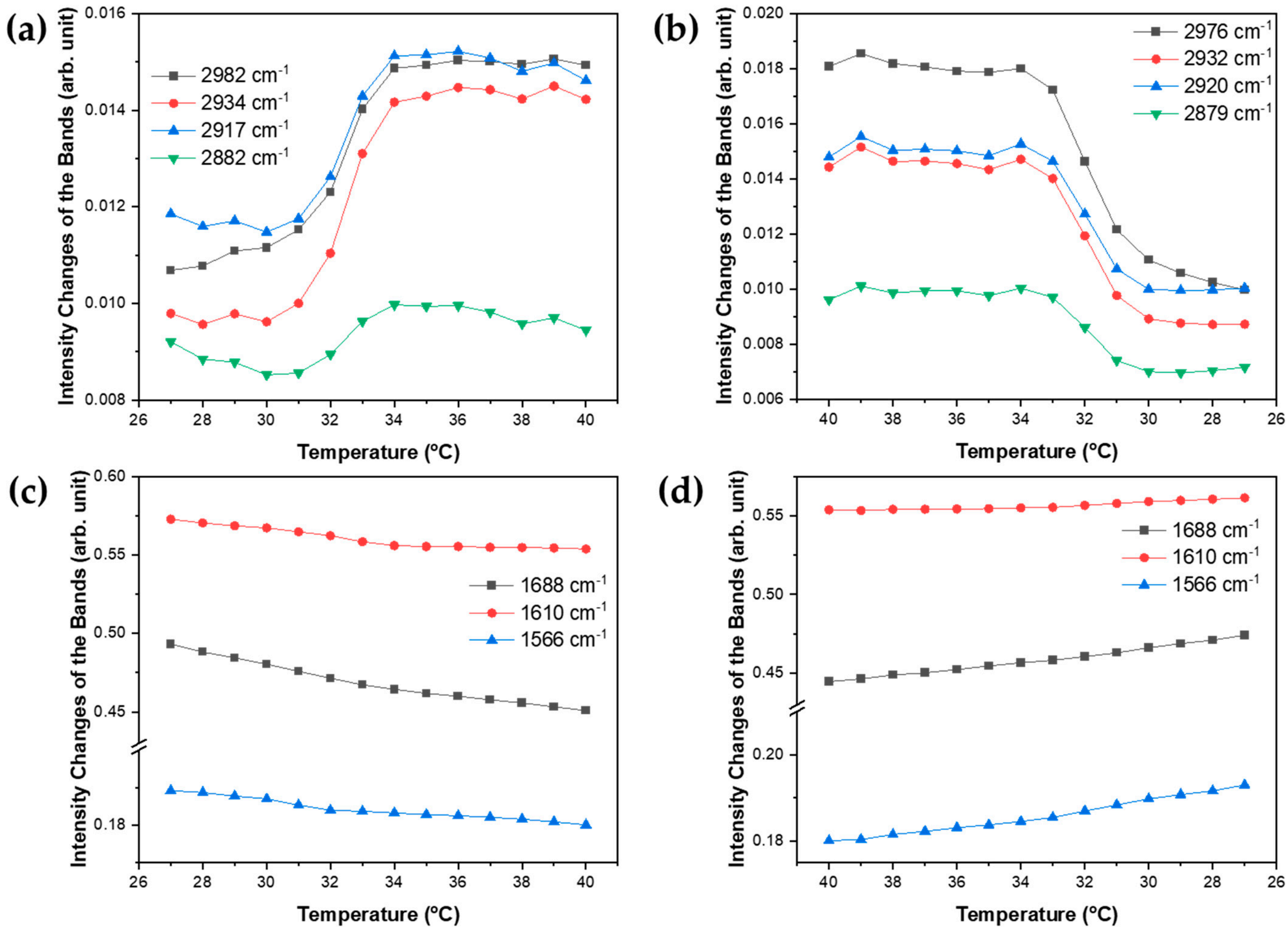

Figure S3. Intensity changes of the bands in two spectral regions (a,b: 3010-2850 and c,d: 1800-1480 cm^{-1}) of P(NPAAm-*co*-AAc) hydrogel at pH2 during the heating (a, c) and cooling (b, d) processes.

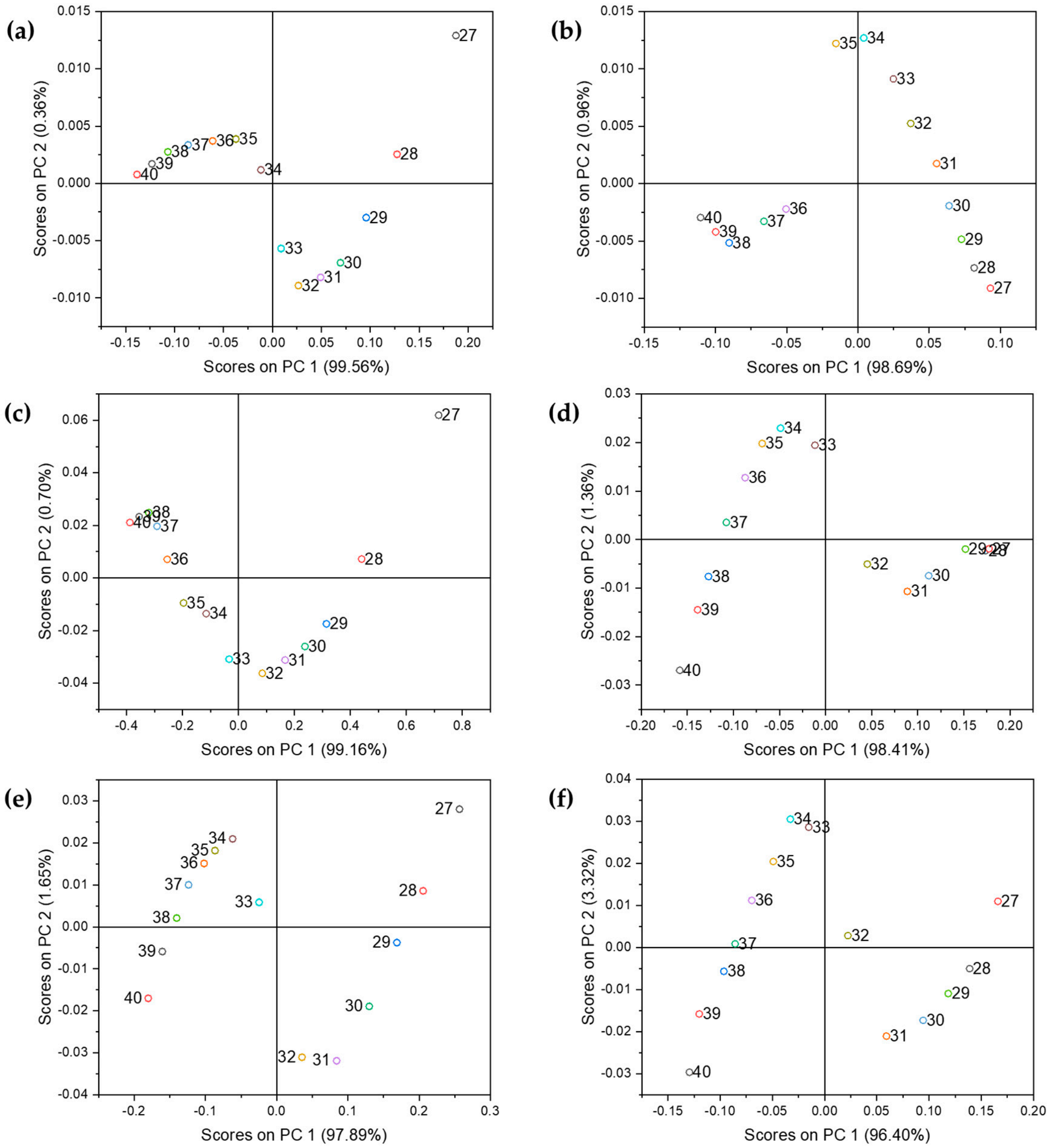

Figure S4. Score plots of the first two PCs of P(NiPAAm-*co*-AAc) hydrogel at pH4 (a,b), 3 (c,d), and 2 (e,f) during the heating (a,c,e) and cooling (b,d,f) processes.
